# Effect of Exercise Training on Quality of Life, Symptoms, and Functional Status in Advanced-Stage Lung Cancer Patients: A Systematic Review

**DOI:** 10.3390/clinpract13030065

**Published:** 2023-06-13

**Authors:** Tena Nguyen, Katharine Tracy, Asad Ullah, Nagla Abdel Karim

**Affiliations:** 1 Medical College of Georgia, Augusta University, Augusta, GA 30904, USA; 2 Department of Pathology, Immunology and Microbiology, Vanderbilt University Medical Center, Nashville, TN 37232, USA; 3 Inova Schar Cancer Institute, Department of Medicine, University of Virginia, Fairfax, VA 22031, USA

**Keywords:** advanced-stage lung cancer, quality of life, functional status, individualized management

## Abstract

Advanced-stage lung cancer (LC) causes significant morbidity and impacts patients’ quality of life (QoL). Exercise has been proven to be safe, feasible, and beneficial for symptom reduction and QoL improvement in many types of cancers, but research is limited in advanced-stage LC patients. This systematic review evaluates the effect of exercise interventions on the symptoms and QoL in patients with advanced-stage LC. Twelve prospective studies (744 participants) were included, evaluating different combinations of exercises and training such as aerobics, tai chi, strength, inspiratory muscle training, and relaxation. Studies found outcomes including but not limited to improved QoL, symptom burden, psychosocial health, functional status, and physical function. The results of this review support that exercise is safe and feasible with evidence supporting improved QoL and symptom mitigation. Integration of exercise should be considered in the individualized management of advanced-stage LC patients under the guidance of their healthcare providers.

## 1. Background

Lung cancer remains the leading cause of cancer-related death in the United States and makes up 12–13% of all cancer diagnoses, second to prostate cancer in men and breast cancer in women [1]. The five-year survival rate of 22.9% is in large part due to the advanced stage of most lung cancer upon detection [2,3]. Increasing awareness of lung cancer and adoption of low-dose CT screening has led to a higher percentage of diagnoses made at stage I and better mortality rates [4,5]. Trends now indicate an increasing prevalence of lung cancer due to earlier detection and decreases in mortality [3]. One study based in Denmark predicted lung cancer prevalence in their country will double from 2015 to 2030, and incidence will plateau [3]. The rates of incidence and mortality vary greatly across the globe, but the general trend of a decrease in incidence and mortality for men and a stabilization for women is seen in most countries [6]. Worldwide, more people will be living with a cancer diagnosis and will face the sequelae of symptoms that follow the disease and its treatment.

The most common symptoms reported by advanced-stage lung cancer patients include fatigue, anorexia, shortness of breath, and pain; shortness of breath, bleeding, and fatigue are cited as the symptoms most dreaded by patients [7,8]. Some research has shown these symptoms are experienced by over 90% of patients, greatly impacting their QoL and ability to carry out daily tasks [9]. One study found that dyspnea and fatigue alone interfered with at least one daily task for over 50% of advanced-stage lung cancer patients in addition to interfering with physical activity and work [10]. Aside from physical and functional impairment, studies have seen significant increases in rates of anxiety and depression with one study reporting that 28.9% of patients were clinically depressed [11]. Another study found that at least 20% of patients reported significant anxiety while almost 25% were not able to meet family needs [12]. Comorbidities also contribute to decreased QoL and worsened prognosis [13,14]. This finding is substantial considering studies have found that 32.2% of people diagnosed with lung cancer have at least one comorbidity [15]. With such a heavy symptom burden, it is important to investigate possible methods of symptom relief to improve the QoL for these patients.

Exercise as one of the adjuvant treatments for cancer patients is now recommended by the American College of Sports Medicine; however, guidelines specific and generalizable to lung cancer patients are not in place [16]. Most research in the field focuses on breast, prostate, colon, gynecologic, and hematological cancer rather than lung cancer [17]. Recent studies have shown that exercise is not only safe and feasible in lung cancer patients but may also be an effective way to decrease symptom burden and improve physical functioning [18]. However, many variables need to be addressed to fully understand the impact of exercise on lung cancer patients. There are many different types of exercise, with varying intensities from breathing-centered workouts to full-body aerobics. Furthermore, the setting of the exercise intervention is another factor to be considered. Studies have investigated at-home programs, group settings, individualized programs, professional-led, and self-led practice.

Previous systematic reviews have looked at the use of exercise in lung cancer patients. For instance, Codima et al., 2021 [19] found that exercise is effective in improving symptoms and QoL, specifically the use of resistance training in combination with high-intensity interval aerobic training after lung resection. Similarly, Cavalheri et al., 2019 [20] also supported the use of exercise in the postoperative management of patients with non-small-cell lung cancer (NSCLC). Interestingly, Gravier et al., 2021 [21], who conducted a systematic review and meta-analysis, concluded exercise prior to lung resection reduced postoperative complications and hospital length of stay. These studies show promise in the use of exercise in the management of lung cancer but have been focused on earlier stages, which are more responsive to lung resection than advanced stages [22].

In other words, although there is a solid increase in the amount of literature being produced on the effect of exercise on lung cancer, fewer studies are focusing on advanced stages. The exclusion of advanced-stage lung cancer patients in exercise intervention studies may be due to questions regarding the ability of this specific group to endure the prescribed exercise regimen [23]. Concerns include but are not limited to increased symptom burden, treatment side effects, and declining life expectancy in those with advanced disease [23]. However, research has begun to show that exercise may be safe and feasible in advanced cancer patients [23]. Therefore, we aim to evaluate the effect of various exercise interventions on QoL, symptoms, and functional status in patients with advanced-stage lung cancer.

## 2. Methods

The review was conducted and reported by the Preferred Reporting Items for Systematic Reviews and Meta-Analyses (PRISMA) guidelines.

### 2.1. Eligibility Criteria

#### 2.1.1. Types of Studies

Prospective studies published in English between January 2012 to March 2022 were considered eligible. Due to missing or lack of extractable data, study protocols and conference abstracts were excluded from the review.

#### 2.1.2. Participants

Participants were defined as advanced lung cancer patients, diagnosed with either non-small-cell lung cancer (NSCLC) stage III–IV and/or limited disease (LD) or extensive (ED) small-cell lung cancer (SCLC). Studies including other stages (I–II) were included if >70% of the patient population had advanced-stage lung cancer. Studies lacking lung cancer stage differentiation or mixed-cancer studies were excluded from the review.

#### 2.1.3. Intervention and Comparison

The intervention was any exercise intervention or deliberate physical exertion. The intervention could take place at any point in time relative to treatment (i.e., chemotherapy, radiation therapy, or surgical resection). Multimodal interventions combined with exercise regimens were also included in the review. The studies included in the review had various control arms. Comparison control groups were defined as either usual care without exercise intervention, usual care with educational resources, or a different exercise intervention.

#### 2.1.4. Outcomes

Studies assessing QoL, symptoms, and/or any functional status were included in the review.

### 2.2. Search Strategy

PubMed, MEDLINE, and Cochrane were searched for eligible prospective studies published between January 2012 to March 2022. Cochrane previously recommended the use of MEDLINE for searching reviews, whereas a previous literature found that PubMed had a higher sensitivity then MEDLINE; therefore, both databases were included along with Cochrane in the literature search [24,25]. Further details regarding the search strategy and key terms for each database are provided in the supplemental section of the review.

### 2.3. Study Selection

Studies searched across all databases were compiled into Endnote, a reference management application [26]. Endnote was used to detect and remove duplicate articles [26]. Articles were screened by title and abstract to remove inapplicable entries. Full-text articles were then subjected to review to assess eligibility and identify if studies were compliant with the predefined inclusion and exclusion criteria. Multiple reports using the same study cohort were included in the review if the article was examining different outcome assessments. After the full-text screen, two reviewers discussed the eligibility of the prospective studies and refined the inclusion criteria. For the article to be included in the review, both reviewers had to agree.

### 2.4. Bias Assessment

Two reviewers using version 2 of the Cochrane Risk-of-Bias (RoB 2) tool for randomized trials assessed the risk of bias and quality of the studies [27]. The five categories evaluated using RoB 2 were as follows: bias arising from the randomization process, bias due to deviations from intended intervention, bias in the measurement of the outcome, and bias in the selection of the reported result. The risk of bias due to deviations from intended intervention was evaluated by two components: bias due to assignment to intervention and bias due to adhering to intervention [27]. For each category, each study was assigned low risk, moderate risk, or high risk based on the criteria outlined [27].

### 2.5. Data Extraction

Following the RoB 2 bias assessment, two reviewers independently used a data extraction form in Excel to extract relevant data and information from the selected eligible studies. The information included in the data extraction was as follows: patient demographics, study location, reported adverse outcomes, the feasibility of the intervention, patient adherence, assessment of outcomes, and results. Descriptions of the intervention and control groups were also included, such as type of exercise, supplementary intervention, as well as length, duration, and timing of intervention about usual anti-neoplastic treatment. Only data that could be extracted from published studies were included in the review. After the initial data extraction, two reviewers discussed the included articles and resolved any disagreements that arose.

## 3. Results

### 3.1. Study Search and Selection

**Figure 1:**
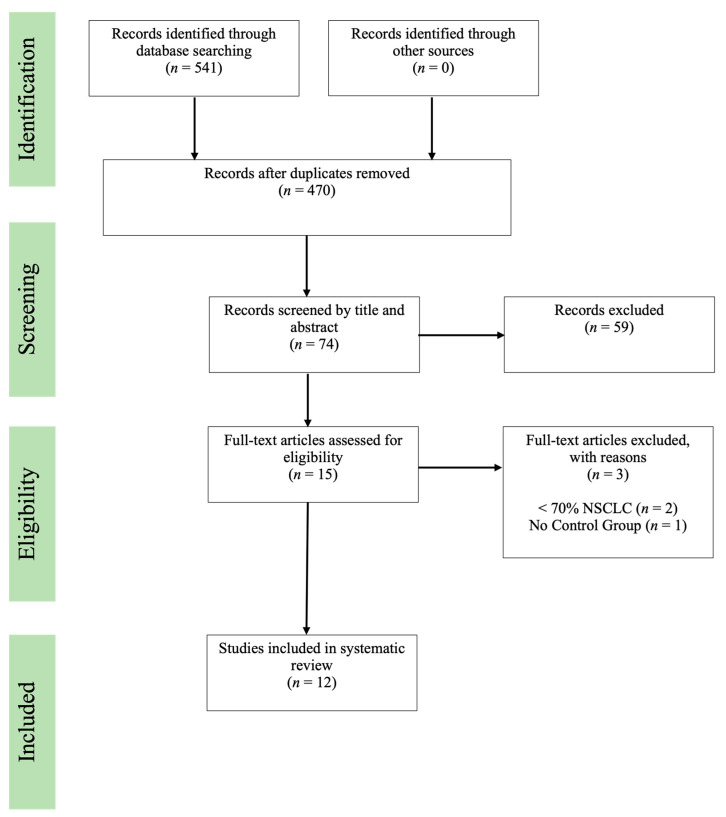
Preferred Reported Items for Systematic Reviews and Meta-Analysis (PRISMA) flow chart. Depicting the flow of the literature search conducted to identify and select the studies included in the systematic review according to PRISMA guidelines.

Literature search and study selection were conducted according to PRISMA guidelines (Figure 1). The search returned 541 studies across three databases. After duplicate records were removed, 470 distinct studies were screened for eligibility by abstract and title. A total of 74 studies were subjected to full-text screening. A total of 15 studies were deemed eligible for further in-depth, full-text review to determine if the study met all defined inclusion criteria. During this stage, three studies were excluded due to <70% of the population sample not having advanced-stage lung cancer (*n* = 2) and absent control group (*n* = 1). Two studies reported findings from the same trial but illustrated independent outcomes in each publication that were included [28,29]. Therefore, a total of 12 prospective studies were included in the review.

### 3.2. Bias Assessment

The quality of the prospective studies selected demonstrated an overall low risk of bias for three studies [28,29,30], overall moderate risk of bias for eight studies [31,32,33,34,35,36,37,38], and overall high risk of bias for one study [39] (Table 1). All 12 studies reported a distinct random allocation sequence. Of the 12 studies, 11 studies reported no significant baseline imbalances. Due to the nature of the intervention of interest, the blinding of participants was improbable. One study reported the exclusion of two participants after baseline testing due to disease progression [32]. The two participants were already allocated to the control group before their exclusion from the study. Another study disclosed the reallocation of three participants after the initial randomization process [33]. The authors stated that two participants, originally allocated to the control group, were motivated to partake in the exercise training, and one participant, originally allocated to the exercise intervention group (IG), wished to be in the control group (CG) due to the far living distance from the clinic [33].

### 3.3. Description of Studies

#### 3.3.1. Patient Demographics

Patient demographics from the studies included in the review are described in Table 2. The review included 744 participants with population samples ranging from 15 participants to 218 participants. Participants were patients diagnosed with advanced-stage lung cancer. The staging distribution varied between studies, with combinations of Stage III–IV NSCLC/SCLC and ED/LD SCLC. One study’s population pool was composed of >70% participants with advanced-stage lung cancer, defined as NSCLC stage III–IV, and advanced thoracic malignancies [37]. Another study included a population with >88% of participants diagnosed with a mix of stage III–IV SCLC or NSCLC [36].

#### 3.3.2. Timing of Antineoplastic Therapy with Regard to Exercise Intervention

The review included studies that evaluated the effects of exercise intervention during or after cancer treatment, as well as in groups that were not receiving treatment at the time of intervention. Most articles included chemotherapy as a form of concomitant cancer treatment. One article specifically included patients undergoing targeted EGFR inhibitor therapy [33]. Other forms of cancer treatments are depicted in Table 2, including combinations of chemotherapy, radiotherapy, targeted therapy, or surgery.

#### 3.3.3. Patient Performance Status

Performance status (PS) is an important factor for clinicians to determine the prognosis, treatment, and management of cancer patients [40]. PS is commonly used to evaluate a patient’s functional ability to complete daily activities without assistance [40]. Nine studies reported Eastern Cooperative Oncology Group/World Health Organization Performance Status (ECOG/WHO-PS) ranging from 0 to 3 [28,29,31,32,33,34,35,36,38]. Two studies recorded PS according to the Karnofsky Scale (KPS) [30,39]. One study included patients with a KPS score ≤ 80 [30]. Another study included patients with a KPS score > 50 [39]. One study did not report performance status [37].

#### 3.3.4. Characteristics of Exercise Intervention

Exercise interventions varied among studies regarding the type of physical activity, length of intervention, frequency, and duration of each session. Five studies’ intervention of interest was aerobic exercise alone [31,32,33,36,38]. Four studies evaluated a mixed exercise regimen including a combination of aerobics, strength, relaxation, and/or endurance [28,29,35,39]. Two studies looked at relaxation exercises and inspiratory muscle training [34,37]. One study conducted a three-arm study comparing two forms of aerobic exercise combined with strength training and tai chi [30]. In addition, one study investigated tai chi alone as an exercise intervention [36]. Two studies evaluated the length of intervention based on three rounds of chemotherapy [34,39]. Overall, the length of the interventions varied from 4 weeks to 12 weeks. The frequency and duration of intervention also differed as shown in Table 3.

Most studies incorporated personalized exercise regimens for each individual based on interest and/or baseline fitness [28,29,31,32,33,35,38,39]. Furthermore, the setting of the exercise interventions differed in terms of location, supervision, or classes. For instance, exercise intervention occurred in the hospital and/or outpatient clinic for six studies [28,29,31,33,35,39], at home for one study [38], and another study [30] took place at a gym class. Exercise interventions in four studies [31,34,36,37] occurred in both a supervised, hospital setting and unsupervised at home or in the community, whereas two other studies [30,35] evaluated exercise interventions in a structured group setting.

### 3.4. Outcomes of Studies

Results of the reviewed studies are included in Table 2. Five broad categories including QoL, symptoms, anxiety/depression, functional status, and physical ability were distinguished to summarize the findings for the review.

#### 3.4.1. Symptoms

All the analyzed studies included data on lung cancer and treatment-related symptoms and symptom management. Five studies found significant improvement for the IG in symptoms including fatigue, dyspnea, hemoptysis, arm and shoulder pain, peripheral neuropathy, cognitive function, vigor, and perception of dyspnea [33,34,36,39].

Additionally, satisfaction with the management of dyspnea was improved in the IG [37]. While most significant findings related to the improvement of symptoms were in the IG, one study found a significant increase in breathlessness and fatigue in the CG, but not in the IG [37].

#### 3.4.2. Quality of Life

Nine out of the twelve included studies directly investigated QoL using European Organization for Research and Treatment of Cancer Quality of Life Core Questionnaire and corresponding lung cancer-specific module (EORTC-QLQ-C30), Functional Assessment of Cancer Therapy-Lung (FACT-L), Strategies Used by People to Promote Health (SUPPH), and St. George’s Respiratory Questionnaire (SGRQ) questionnaires [29,30,31,32,33,34,35,38,39]. Unlike the SUPPH and the SGRQ, the EORTC-QLQ-C30 and the FACT-L are QoL assessments used among cancer patients, with an additional module specifically designed for lung cancer patients [41,42].

Of these studies, 1/9 found a statistically significant increase in SUPPH scores for the IG, indicating an increased ability for patients to cope with stress, make decisions, and exhibit positive behaviors [34]. This study also associated a decrease in symptom frequency and severity with a subsequent increase in self-efficacy as was seen in the IG. It was also found that the CG had a significant decrease in social wellbeing throughout the intervention, in the end creating a significant difference in the social well-being of the IG compared to the control [35]. Another study also found significant differences between the interventional and CG with improvement in IG with respect to the role functioning domain of the EORTC-QLQ-C30 [38].

#### 3.4.3. Anxiety and Depression

Six of the twelve studies assessed for psychosocial health, anxiety, and depression, using the Hospital Anxiety and Depression Scale (HADS-9), Short General Health Questionnaire (GHQ-12), or Memorial Symptom Assessment Scale-Psychological Symptom Distress Score (MSAS-PSYCH) questionnaires [30,31,32,34,35,37]. Of these studies, 3/6 found statistically significant improvement in both anxiety and depression for patients in the IG [30,34,37]. Significant increases in anxiety and depression were seen in the CG by two studies [35,37].

#### 3.4.4. Functional Status

Four of the twelve studies evaluated functional status using Activities of Daily Living (ADL), Instrumental Activities of Daily Living (iADL), or the Fullerton test [28,30,31,39]. Several studies found that functional tasks including the sit up and go, 30 s sit to stand, chair stand, and arm curl tests were significantly improved in the IG [28,30]. In addition, it was found that the CG had a decrease in the chair sit and reach up and go tests, with the difference between intervention and control groups being significant for the get-up and go test [28]. Another study found the overall effect on the patient’s independence in carrying out activities of daily living was significantly improved in the IG [39].

#### 3.4.5. Physical Functioning

Six studies investigated physical function using the six-minute walking distance/test (6MWD), VO2 peak, predicted VO2 peak, and 1-repetition maximum (1RM) [28,30,32,33,35,39]. A significant increase in the 6MWD test was seen in three of the included studies [28,30,39]. Additionally, studies found an increase in physical strength, balance, stair walking ability, and general physical function for the IG [30,35,39]. VO2 peak and predicted VO2 peak were also seen to be significantly increased in the IG [33].

#### 3.4.6. Spirometry

In addition to the above-mentioned categories, four studies evaluated pulmonary function using spirometry [28,32,35,37]. One of these studies found significant improvement in FEV1, FVC, and FEV1/FVC in the IG compared to baseline [28].

#### 3.4.7. Biological Biomarkers

Three studies evaluated biological biomarkers [31,33,38]. One study measured insulin, leptin, C-reactive protein (CRP), soluble programmable death protein (sPD-1), and soluble programmable death ligand (sPD-L1) [38]. The study found no significant differences except between-group changes in sPD-1 (*p* < 0.001) with improved levels in the exercise group and decreases in the CG. Another study evaluated blood samples for the modified Glasgow Prognostic Score (CRP, protein, and albumin), cytokines, and insulin-like growth factors (IGF) [31]. However, the study did not report any significant findings. Blood analysis in one study examined homeostatic model assessment of insulin resistance (HOMA-IR) and highly sensitive CRP (Hs-CRP) [33]. The study also noted six participants in the study declined bloodwork, but the data collected from participating patients, HOMA-IR, and Hs-CRP were unchanged in both the IG and CG [33].

## 4. Discussion

This review intended to evaluate the QoL, symptoms, and functional status in advanced lung cancer patients participating in an exercise regimen. Treatment and management in advanced lung cancer patients aim to prolong patient survival as well as maintain or improve their QoL due to the debilitating effects of progressing lung cancer and treatment [43]. Most of the studies included in the review evaluated QoL as an outcome. One study looked at individualized walking goals and found significant differences between the IG and CG in the role functioning domain of the EORTC-QLQ-C30, in favor of the IG [38]. These findings are supported by previous studies in that the QoL in lung cancer patients can be improved by exercise [44,45].

Quality of life also encompasses psychosocial components such as anxiety and depression [46]. A previous study demonstrated that lung cancer patients experienced the highest levels of stress and anxiety in comparison to other types of cancer [47]. Moreover, depression is a predictor of increased mortality in recently diagnosed lung cancer patients [48]. Two studies using the HADS found that the IG had improved anxiety and depression compared to baseline than the CG [30,37]. Two other studies also found that the CG had significantly worse anxiety and depression when compared to baseline [35,37]. The deterioration in the psychosocial health of the CG is crucial in understanding the role that exercise may play in maintaining anxiety and depression levels in patients with advanced lung cancer.

Likewise, in patients with advanced-stage cancer, exercise was previously proven to be effective in decreasing cancer symptoms such as fatigue and dyspnea [43]. Five studies noted a significant decrease in cancer symptoms in the IG [33,34,35,36,39]. One study looked at the effects of inspiratory muscle therapy and found that the CG experienced a significant difference in the worsening of symptoms such as mean breathlessness and distress in comparison to the IG [37]. These findings spark a further investigation into the integration of respiratory exercises in the clinical management of advanced lung cancer patients for the alleviation of cancer symptoms.

The review included studies utilizing several types of exercises, settings, and participant interaction with others. Aerobic exercises broadly include physical activity that increases heart rate and the body’s oxygen demand (i.e., walking, stationary bike, etc.). In addition to aerobic exercises, respiratory exercises, strength training, and tai chi all showed significant results in this review. General recommendations for cancer patients include the primarily aerobic exercise of moderate intensity yet the definitive superiority of one exercise modality over another is still uncertain in advanced-stage lung cancer patients [16]. Interestingly, the studies in this review that only utilized breathing exercises found significant improvement in psychosocial health as well as improvement in QoL [34,37]. These findings indicate the addition of respiratory exercises such as inspiratory resistance training and controlled relaxation/breathing may be beneficial to advanced-stage lung cancer patients or could serve as a standalone exercise intervention for deconditioned patients who cannot physically participate in vigorous aerobic exercise.

Tai chi was found to be an effective exercise intervention and was even found to be superior to aerobic training in decreasing anxiety and depression in one study [30]. In this review, tai chi was included only in studies originating in China, reflecting the cultural ties to the specific exercise. This exercise may have advanced feasibility and acceptance in people who associate tai chi with their culture. Though our review did not include other mind-body exercises such as yoga, one study found that lung cancer patients had improvement in QoL and decreased fatigue with yoga intervention [49].

The setting was also variable among the studies and may influence the efficacy of the intervention. At-home programs may be more feasible but may inevitably result in loss of accountability and a drop in adherence. Evidence suggests that adherence to at-home exercise programs may be related to self-efficacy at baseline and may thus be a better option for patients with high self-efficacy and motivation [50]. In contrast, another study included in this review that utilized group exercise found a significant increase in the well-being of patients [35]. Other studies support this and report group cohesion, autonomy, and social support for group exercise in lung cancer patients [51,52]. For patients with limited social support, group exercise may be one way to not only help improve symptom burden but also increase social wellbeing and support.

In this review, we see similar results across geographical regions representing four major continents. This diversity in study demographic indicates that the benefits of exercise as treatment are likely generalizable to advanced-stage lung cancer patients worldwide.

Our findings support previous studies in the assessment that exercise is a safe and feasible option for advanced lung cancer patients. No serious adverse events were reported as a result of exercise interventions in the selected studies, though adverse events deemed unrelated did occur during the timeline of the program [30,31,32,38,39]. The range of attrition rates for the selected studies was broad, ranging from 13% to 67% [28,29,30,31,32,33,35,39]. A common cause of attrition from the study was the patient’s worsening condition or death due to lung cancer progression. Also common was a lack of motivation to finish the program, likely due to increasing fatigue from treatment. The average reported adherence for the studies included in the review is 65.79% with rates ranging from 21% to 88.1%. Though adherence is an important component to determine the validity of a study, the study group population must be taken into consideration. Advanced-stage lung cancer patients often experience symptoms that interfere with daily functioning, including participation in exercise. This calls into question the impact of exercise intensity on patient adherence and feasibility. Quantifiable information on exercise intensity was limited in our selected studies but is a factor to consider in future studies.

This review presents several limitations. It is difficult to distinguish the effect of the medical treatment from the exercise intervention in the design of these studies, especially since treatment options have evolved rapidly in recent years. Most of the included studies had small population samples, decreasing the statistical power of their findings. Larger powered randomized controlled trials are needed for further in-depth conclusive results evaluating the effect of exercise on outcomes in advanced lung cancer. Most of the studies screened for performance status as part of their study inclusion criteria. For instance, 5/9 studies that evaluated PS only included patients with a PS of 0-1, reducing the generalizability of this information to patients with PS 2-3 [28,29,32,33,39]. With the support that exercise is an option for advanced lung cancer patients, future studies should evaluate the impact of individualized exercise in patients with PS 2-3 as well. Therefore, another limitation to our study was the lack of generalizability through the exclusion of participants with more debilitating forms of the disease. Another limitation is that among the studies included in our review, the implementation of exercise as a part of the treatment regimen varied in the setting, duration, and timing of exercise during the different stages of treatment. Moreover, the included studies did account for adverse outcomes; however, due to design of the studies, the side effects of the treatment regimen may not be solely attributable to solely exercise or adjunctive treatment (i.e., chemotherapy, radiotherapy, targeted therapy, etc.) or may be multifactorial. Furthermore, only 12 studies were included in this review, indicating the lack of and need for more research to be performed assessing the effects of exercise in patients with advanced stages of lung cancer.

Studies investigating the effects of exercise intervention in advanced-stage lung cancer patients are currently underway. One clinical trial is planning to evaluate the effect of tai chi and aerobic interventions on sleep disturbance, anxiety, depression, and fatigue in advanced-stage lung cancer patients [53] Another clinical trial is targeting the effectiveness of physical therapy and muscle relaxation on improving symptom burden and QoL [54]. However, further exploration of exercise in advanced lung cancer would provide clinical implications in its management, namely in providing more safe and feasible alternative exercises that can be individualized to the interests and needs of each patient.

## 5. Conclusions

Exercise in advanced-stage lung cancer patients is not only safe and feasible, but also shows evidence in improving QoL and symptom burden. Different forms of exercise have proven to be effective, including aerobics, tai chi, and respiratory training. Intervention for patients should be individualized based on physical fitness and health to maximize QoL and symptom management. This systematic review aims to provide clinicians insight on exercise regimens with differing settings, duration, and timing of treatment and, consequently, help providers identify and further personalize effective management in the alleviation of symptoms and QoL for each patient with advanced-stage lung cancer with consideration towards factors such as patient preference, feasibility, physical health, and emotional well-being. Future research is needed to develop guidelines for healthcare providers to follow when recommending exercise as a management tool for these patients.

## Figures and Tables

**Table 1:** Risk of bias assessment of included studies using version 2 of the Cochrane Risk-of-Bias (RoB 2) Tool for Randomized Trials.

Category/ Study	Risk of Bias from Randomization Process	Effect of Assignment to Intervention	Effect of Adhering to Intervention	Risk of Bias due to Missing Outcome Data	Risk of Bias in Measurement of the Outcome	Risk of Bias in Selection of the Reported Result	Overall Risk of Bias
Rutkowska et al., 2019 [28]	L	L	L	L	L	L	L
Rutkowska et al., 2021 [29]	L	L	L	L	L	L	L
Cheung et al., 2021 [30]	L	L	L	L	L	L	L
Dhillon et al., 2017 [31]	L	L	L	L	M	L	M
Egegaard et al., 2019 [32]	L	M	L	L	M	L	M
Hwang et al., 2012 [33]	L	M	L	L	M	L	M
Kirca et al., 2021 [34]	L	L	L	L	M	L	M
Quist et al., 2020 [35]	L	L	M	M	L	L	M
Zhang et al., 2016 [36]	L	L	L	L	M	L	M
Molassiotis et al., 2015 [37]	L	L	L	L	M	L	M
Bade et al., 2021 [38]	L	L	M	L	M	L	M
Henke et al., 2014 [39]	M	M	L	L	M	L	H

Bias was assessed as low risk (L), moderate risk (M), or high risk (H).

**Table 2:** Patient demographics of included studies.

Study	Country	Study Population	Mean Age (Years)	Gender (*n*, %)	Cancer Stage(s)	Performance Status	Treatment Status
Rutkowska et al., 2019 [28]	Poland	*n* = 30	IG 59.1 ± 6.8 CG 61.3 ± 8.8	F (*n* = 3, 10%) M (*n* = 27, 90%)	NSCLC IIIb–IV; disqualified from surgery	WHO-PS 0-1	During in-hospital chemotherapy
Rutkowska et al., 2021 [29]	Poland	*n* = 26	IG 60.4 ± 7.2 CG 62.2 ± 9.0	F (*n* = 2, 7.7%) M (*n* = 24, 92.3%)	NSCLC IIIb–IV; disqualified from surgery	ECOG-PS 0-1	During in-hospital chemotherapy
Cheung et al., 2021 [30]	Hong Kong	*n* = 30	AG 61.0 ± 12.12 TC 61.11 ± 7.01 CG 58.36 ± 9.32	F (*n* = 14, 46.7%) M (*n* = 16, 53.3%)	NSCLC IIIb–IV	KPS score ≤ 80	Chemotherapy, radiotherapy, targeted therapy, no treatment
Dhillon et al., 2017 [31]	Australia	*n* = 111	IG 64 (range 38–80) CG 64 (range 34–76)	F (*n* = 50, 45%) M (*n* = 61, 55%)	NSCLC III–IV LS SCLC	ECOG-PS 0-2	Chemotherapy, chemotherapy + targeted agent, targeted therapy only, no active treatment
Egegaard et al., 2019 [32]	Denmark	*n* = 15	IG 64 ± 5.8 CG 65 ± 4.7	F (*n* = 10, 66.7%) M (*n* = 5, 33.3%)	NSCLC III–IV	WHO-PS 0-1	During chemoradiotherapy
Hwang et al., 2012 [33]	Taiwan	*n* = 24	IG 60.4 ± 7.2 CG 62.2 ± 9.0	F (*n* = 12, 50%) M (*n* = 12, 50%)	NSCLC III–IV	ECOG-PS 0-1	During targeted therapy (EGFR inhibitors) for at least 4 weeks
Kirca et al., 2021 [34]	Turkey	*n* = 84	IG 60.4 ± 7.2 CG 62.2 ± 9.0	F (*n* = 69, 82.1%) M (*n* = 15, 17.9%)	NSCLC/SCLC III–IV	ECOG-PS 0-2	During chemotherapy
Quist et al., 2020 [35]	Denmark	*n* = 218	OG 64.4 ± 8.5 IG 65.2 ± 8.2 CG 63.5 ± 8.7	F (*n* = 111, 50.9%) M (*n* = 107, 49.1%)	NSCLC III–IV SCLC LS/ES	WHO-PS 0-2	During chemotherapy
Zhang et al., 2016 [36]	China	*n* = 91	OG 62.8	F (*n* = 23, 25.3%) M (*n* = 68, 74.7%)	88% NSCLC/SCLC III–IV	ECOG-PS 0-3	During cisplatin-based chemotherapy
Molassiotis et al., 2015 [37]	United Kingdom, Cyprus	*n* = 46	Not reported	F (*n* = 9, 19.6%) M (*n* = 37, 80.4%)	70% NSCLC III–IV and advanced thoracic malignancy	Not reported	Post chemotherapy only, radiotherapy only, chemoradiotherapy, surgery only, surgery + chemotherapy, surgery + chemotherapy + radiotherapy
Bade et al., 2021 [38]	USA	*n* = 40	OG 64.88 ± 8.69 IG 66.55 ± 7.28 CG 63.20 ± 9.80	F (*n* = 30, 75%) M (*n* = 10, 25%)	NSCLC III–IV	ECOG-PS 0-1	Chemotherapy, immunotherapy, targeted therapy, post-treatment
Henke et al., 2014 [39]	Germany	*n* = 29	Not reported	Not reported	NSCLC/SCLC III–IV	KPS score > 50	During in-patient palliative platinum-based chemotherapy

Intervention Group (IG), Control Group (CG), Overall Group (OG), Aerobic Group (AG), Tai Chi Group (TG), Female (F), Male (M), Non-small-cell lung cancer (NSCLC), small-cell lung cancer (SCLC), limited stage (LS), extensive stage (ES), Eastern Cooperative Oncology Group Performance Status (ECOG-PS), World Health Organization Performance Status (WHO-PS), Karnofsky Performance Scale Index (KPS).

**Table 3:** Study design and characteristics of included studies.

Study	Study Design	Setting	Type/ Frequency/Length of Intervention	Assessment of Outcomes	Results	Adherence	Adverse Outcomes
Rutkowska et al., 2019 [28]	RCT	Supervised, hospital setting	Type: endurance, breathing, weight, and fitness training Frequency: 5 times per week for 30–45 min each session Length: 4 weeks in 2-week cycles; 6-week period between assessments	6MWT, spirometry, mMRC, BDS, Fullerton Test	Statistically significant increase in IG when compared to baseline with 6MWD (*p* = 0.01), up-and-go test (*p* = 0.01), chair stand (*p* = 0.01), and arm curl (*p* = 0.001). CG showed significant decrease in the chair sit and reach and up-and-go tests. The up-and go-tests between groups showed statistical significance. Spirometry also improved significantly with FEV1 % predicted, FVC % predicted, and FEV1/FVC. No significant improvements in CG. mMRC showed no statistical significance in dyspnea improvement, but BDS showed significant improvement in perception of dyspnea (*p* = 0.04) in IG. No significant changes in CG.	25% attrition rate.	No adverse outcomes reported.
Rutkowska et al., 2021 [29]	RCT	Supervised, hospital setting	Type: endurance, breathing, weight, and fitness training Frequency: 5 times per week Length: 4 weeks in 2-week cycles; 6-week period between assessments	SGRQ, SF-36, FACT-L	No statistically significant changes in SGRQ measuring QoL. However, intermediate effect size noted in symptom domain and impact of life domain, favoring IG. No statistically significant changes in IG for FACT-L with significant decrease in the CG’s physical wellbeing (*p* < 0.02). No significant changes in either group for SF-36.	27% attrition rate.	No adverse outcomes reported.
Cheung et al., 2021 [30]	Assessor blinded, pilot feasibility RCT	Supervised aerobic or tai chi class	Type: aerobic, tai chi Frequency: 60 min 2× per week + 90 min self-practice (total 150 min/week) Length: 12 weeks (3 months)	PSQI, HADS, BFI, EORTC QLQ-C30, QLQ-LC13, physical performance, actigraph, and circadian rhythms (salivary cortisol)	Aerobic group showed statistically significant improvement post-intervention in time up-and-go (−2.26, 95% CI: −4.04, −0.48) and 30 s sit-to-stand tests (4.52, 95% CI: 2.19, 6.85) than the tai chi and control groups. Improvement in anxiety in tai chi group post-intervention (−1.45, 95% CI: −4.62, 1.72), 6-month (−2.13, 95% CI: −5.30, 1.04), and 1-year follow up (−1.98, 95% CI: −5.18, 1.22) relative to baseline. Aerobic and control groups reported smaller improvements. Tai chi showed more improvement in balance (28.25, 95% CI: −37.08, 93.58) and 6MWT (19.42, 95% CI: −44.83, 83.67) than the aerobic group. Control group showed improved anxiety, depression, sleep disturbance, and some aspects of physical performance (up-and-go, sit-to-stand tests).	Aerobic Group: 80% Tai Chi Group: 78%	No adverse events in the tai chi group. One participant in the aerobic group reported lip numbness during class, unrelated to intervention.
Dhillon et al., 2017 [31]	Open labeled RCT	Supervised with exercise provider and unsupervised sessions	Type: aerobic Frequency: 1 h/week Length: 8 weeks	FACT-F, EORTC-QLQ-C30-LC-13, ADL, iADL, GHQ-12, distress thermometer, physical performance FACT-Cognition, PSQI, spirometry, Glasgow Prognostic Score, biological blood samples; Questionnaires: Shortness of Breath, Active, Sedentary Behavior, Social Cognitive Determinants of Exercise	No difference in mean scores between groups in QoL. No significant difference between groups in fatigue, symptoms, dyspnea, stress, anxiety/depression, cognitive symptoms, sleep quality, activities of daily living, physical function, fitness, anthropometric measures, overall survival, Glasgow Prognostic Score, or biological biomarkers.	69% in physical activity component; 75% in behavioral support sessions	No serious reported adverse events. Four participants in IG reported back/muscle soreness that resolved with no treatment. Four other participants had minor adverse events that were resolved with no treatment.
Egegaard et al., 2019 [32]	Feasibility RCT	Supervised, hospital setting	Type: aerobic Frequency: 20 min daily Length: 7 weeks	HADS, FACT-L, IPAQ-L, spirometry, 6MWD	No significant differences between groups for QoL via FACT-L. No significant differences within or between group differences with anxiety and depression. No significant differences between groups in steps, distance, or intensity minutes. No significant differences within or between groups from baseline to post-intervention in any cardiopulmonary endpoints.	88.1% adherence to full exercise participation. No dropouts during intervention.	Two participants were hospitalized during the course due to chemotherapy adverse events. No reported events occurred during the exercise session.
Hwang et al., 2012 [33]	RCT	Supervised, outpatient clinic	Type: high intensity interval aerobic training Frequency: 3 time per week for 30–40 min each session Length: 8 weeks	CPET, NIRS, venous blood sample, isokinetic muscle testing, EORTC QLQ-C30	Significant increase favoring IG found in VO2peak (*p* < 0.005) and %predVO2peak (*p* < 0.05). HOMA-IR and Hs-CRP unchanged in both groups. No between group differences in QoL. IG had a significant decrease in dyspnea and fatigue from baseline (*p* = 0.05).	Mean adherence rate of the exercise group was 71.2%.	No exercise-related adverse outcomes reported.
Kirca et al., 2021 [34]	RCT	Supervised hospital; at home setting	Type: relaxation exercises Frequency: 30 min daily Length: 3 courses of chemotherapy	MSAS, SUPPH, weekly telephone counseling	Statistically significant decrease in mean MSAS-GDI, MSAS-PSYCH and TMSAS in IG. Significant increase in SUPPH scores in IG. No statistically significant change among CG. Self-efficacy increased as frequency and severity of symptoms decreased (*p* = 0.000).	Not reported.	No adverse outcomes reported.
Quist et al., 2020 [35]	RCT	Hospital setting	Type: aerobic, strength, and fitness training Frequency: 1.5 h, 2 times/week Length: 12 weeks	VO2peak, 1RM, 6MWT, spirometry, FACT-L, HADS	No statistically significant difference in aerobic capacity based on VO2peak between IG and CG. Statistically significant increase in strength with leg press (*p* = 0.01), extension (*p* < 0.01), chest press (*p* < 0.01), and lat pull down (*p* = 0.04) in IG. Statistically significant difference between groups with decrease in social wellbeing (*p* = 0.04) and increase in anxiety (*p* = 0.02) and depression (*p* = 0.01) in CG.	44% adherence rate.	No adverse outcomes reported.
Zhang et al., 2016 [36]	RCT	Unsupervised at home or supervised in community	Type: tai chi Frequency: every other day for 1 h sessions Length: 12 weeks	MSFI-SF	Significant decrease in IG when compared to CG in MFSI-SF total score, general fatigue scores, and physical fatigue scores (*p* < 0.05). Significant higher vigor score in IG than CG (*p* < 0.05). No significant differences in emotional or mental subscale. Results were similar at 6 weeks and 12 weeks.	Not reported.	No adverse outcomes reported.
Molassiotis et al., 2015 [37]	Feasibility RCT	Supervised in hospital; home setting	Type: inspiratory muscle training Frequency: 30 min sessions, 5 times/week Length: 12 weeks	CRDQ, MBS, HADS, spirometry	CG was statistically significantly higher than the IG group for the worst breathlessness over the past 24 h (*p* = 0.003 and average breathlessness over the past 24 h (*p* = 0.019). There was increasing distress experienced due to breathlessness in the CG from wk 4 to wk 12 (*p* = 0.035). Compared to the IG group, the mean distress for the CG was significantly higher (*p* = 0.018). Mean scores for coping ability were higher for the IG group at wk 4 (*p* = 0.012) and wk 8 (*p* = 0.023). Satisfaction with management of breathlessness was significantly higher in the IG group at wk 4, wk 8, wk 12 (*p* = 0.02, 0.001, 0.001). Mastery scores were also significantly lower in the CG group at wk 4, wk 8, wk 12 (*p* = 0.015, 0.028, 0.036). For the HADS scores, the IG group was significantly better than measured at baseline (*p* = 0.034) while the CG group was significantly worse than baseline (*p* = 0.026 and *p* = 0.035) as measured at wk 4 and wk 8. The CRDQ scale showed better fatigue scores in the IG group at wk 4, wk 8, wk 12.	Not reported.	No adverse outcomes reported.
Bade et al., 2021 [38]	Pilot open-labeled RCT	Home	Type: aerobic (walking) Length: 12 weeks (3 months)	MMRC Dyspnea Scale, Modifiable Activity Questionnaire, EORTC-QLQ-C30, PHQ-9, cancer biomarkers (insulin, leptin, CRP, sPD-1, sPD-L1)	Both groups reported higher QLQ summary scores though not significant. Significant between group differences in the role functioning domain of EORTC (*p* = 0.02). No statistically significant increase with both groups in MMRC dyspnea scale. Significant between group differences in sPD-1 compared to baseline (*p* < 0.001) with increases in IG and decreases in CG.	Individualized walking goals met in 21% of weeks.	Four serious adverse events unrelated to study (three hospitalizations, one ER visit); two minor adverse events unrelated to study.
Henke et al., 2014 [39]	RCT	Supervised, hospital setting	Type: strength, endurance, and breathing training Frequency/Length: 3 rounds of chemotherapy	ADL, EORTC-QLQ-LC-13, 6MWT, staircase walking, MBS	Statistically significant difference in EORTC-QLQ-C-30-L-13 for physical functioning (*p* = 0.025), hemoptysis (*p* = 0.019), arm/shoulder pain (*p* = 0.048), peripheral neuropathy (*p* = 0.050), cognitive functioning (*p* = 0.050). Significant differences favoring the IG found in ADL (*p* = 0.041), 6MWT, stair walking, strength capacity, patient’s dyspnea perception. Baseline differences in endurance capacity and strength between groups before and after intervention.	Originally started with 46 patients, but only 29 completed the intervention.	Six participants did not complete the trial due to death; otherwise, no reported adverse outcomes.

Randomized Controlled Trial (RCT), Interventional Group (IG), Control Group (CG), Standard Error (SE), Confidence Interval (CI), Emergency Room (ER), Pittsburgh Sleep Quality Index (PSQI), Hospital Anxiety and Depression Scale (HADS-9), Brief Fatigue Inventory (BFI), European Organization for Research and Treatment of Cancer Quality of Life Core Questionnaire and corresponding lung cancer-specific module (EORTC QLQ-C30, QLQ-LC13), rate of perceived exertion (RPE), homeostatic model of insulin resistance (HOMA-IR), high-sensitivity C-reactive protein (Hs-CRP), C-reactive protein (CRP), Soluble programmed death protein 1 (sPD-1), Soluble programmed death protein Ligand-1 (sPD-L1), Multidimensional Fatigue Symptom Inventory-Short Form (MSFI-SF), St. George’s Respiratory Questionnaire (SGRQ), Short Form Health Survey (SF-36), Functional Assessment of Cancer Therapy-Lung (FACT-L), Functional Assessment of Cancer Therapy-Fatigue (FACT-F), Modified Medical Research Council Dyspnea Scale (mMRC), Borg Dyspnea Scale (BDS), repetition maximum (RM), Memorial Symptom Assessment Scale (MSAS), Memorial Symptom Assessment Scale-Global Distress Index (MSAS-GDI), Memorial Symptom Assessment Scale-Psychological Symptom Distress Score (MSAS-PSYCH), Total Memorial Symptom Assessment Scale (TMSAS), Strategies Used by People to Promote Health (SUPPH), Chronic Respiratory Disease Questionnaire (CRDQ), MBS (Modified Borg Scale), 1-Repitition Max (1RM), World Health Organization (WHO), 6-Minute Walk Distance (6MWD), 6-Minute Walk Test (6MWT), Activities of Daily Living (ADL), Instrumental Activities of Daily Living (iADL), Short General Health Questionnaire (GHQ-12), Patient Health Questionnaire (PHQ-9), percentage of predicted VO2peak (%predVO2peak), cardiopulmonary exercise testing (CPET), near-infrared spectroscopy (NIRS), Inspiratory Muscle Training (IMT), Week (Wk), maximum HR (HRmax).

## Data Availability

The data supporting the findings of this study are available within the article and/or in the supplementary information.

## Supplementary Materials

The following supporting information can be downloaded at: https://www.mdpi.com/article/10.3390/clinpract13030065/s1, Table S1: Search Strategy per Database; Table S2: Detailed Intervention and Control Group Description.
