# Supplementary material for: Effect of Exercise Training on Quality of Life, Symptoms, and Functional Status in Advanced-Stage Lung Cancer Patients: A Systematic Review
Source: Clin Pract. 2023 Jun 13;13(3):715–30. doi: 10.3390/clinpract13030065 (PMC10296866; doi:10.3390/clinpract13030065)
Supplement: Supplementary file 1 [file clinpract-13-00065-s001.zip › clinpract-2393203-supplementary.pdf]

Table S1: Search Strategy per Database

PubMed

#	Searches	Results
1	((((("Lung Neoplasms"[Mesh]) OR ("Lung Neoplasms/prevention and control"[Mesh] OR "Lung Neoplasms/rehabilitation"[Mesh])) OR (advanced lung cancer[MeSH Terms])) OR (palliative[MeSH Terms]))	313,831
2	((((quality of life[MeSH Terms]) OR (value of life[MeSH Terms]) OR (qaly[MeSH Terms]) OR (quality of wellbeing[Other Term]) OR (anxiety[MeSH Terms]))OR (fatigue[MeSH Terms])) OR (assessment, patient outcome[MeSH Terms]) OR (assessment, outcome health care[MeSH Terms])))	1,579,011
3	(((aerobic exercise[MeSH Terms]) OR (tai chi[MeSH Terms]) OR (resistance training[MeSH Terms]) OR (yoga[MeSH Terms]) OR (physical activity[MeSH Terms]) OR (physical activities[MeSH Terms]) OR (exercise[MeSH Terms])) OR (dance therapies[MeSH Terms]))	232,137
4	#1 AND #2 AND #3	205
5	#4 AND PUBYEAR > 2012	148
6	Filters: clinical trial, clinical study, randomized controlled trial	50

MEDLINE

Searches	Results
Clinical Queries: advanced lung cancer and exercise<br>Category: therapy<br>Emphasis: sensitive search (broad)	79
PUBYEAR > 2012	62

Cochrane

#	Searches	Results
1	MeSH descriptor: [Lung Neoplasms] explode all trees	8413
2	MeSH descriptor: [Lung Neoplasms] explode all trees and with qualifier(s): [rehabilitation - RH]	45
3	Advanced lung cancer	10,327
4	Pulmonary neoplasm	894
5	Pulmonary cancer	5167
6	Pulmonary neoplasms	1809

7	MeSH descriptor: [Lung Neoplasms] explode all trees and with qualifier(s): [prevention & control - PC]	177
8	#1 OR #2 OR #3 OR #4 OR #5 OR #6 OR #7	19,860
9	MeSH descriptor: [Quality of Life] explode all trees	27,833
10	MeSH descriptor: [Value of Life] explode all trees	33
11	MeSH descriptor: [Quality-Adjusted Life Years] explode all trees	1,337
12	MeSH descriptor: [Anxiety] explode all trees	8,902
13	MeSH descriptor: [Fatigue] explode all trees	4,131
14	MeSH descriptor: [Depression] explode all trees	13,714
15	quality of life	141,471
16	value of life	17,723
17	symptoms	169,637
18	symptom	70,670
19	symptom burden	4,179
20	MeSH descriptor: [Patient Outcome Assessment] explode all trees	1,317
21	#9 OR #10 OR #11 OR #12 OR #13 OR #14 OR #15 OR #16 OR #17 OR #18 OR #19 OR #20	313,373
22	MeSH descriptor: [Exercise] explode all trees	27,794
23	Exercise	113060
24	Exercises	27437
25	physical activity	52598
26	physical activities	15412
27	exercise therapy	49225
28	exercise therapies	3,436
29	aerobic exercise	15,114
30	aerobic	17,716
31	Resistance training	15,065
32	high intensity interval training	4,007
33	Yoga	4,314
34	Dance	1,191
35	Dance therapies	151
36	Dance therapy	560
37	#22 OR #23 OR #24 OR #25 OR #26 OR #27 OR #28 OR #29 OR #30 OR #31 OR #32 OR #33 OR #34 OR #35 OR #36	161,690

38	#8 AND #38 AND #21 with Cochrane Library publication date Between Jan 2012 and Mar 2022, in Cochrane Trials (Word variations have been searched)	370

Table S2: Detailed Intervention and Control Group Description

Study	Intervention and Control Group Description
Rutkowska et al., 2019[21]	IG (N=20): Personalized exercise regimens including warm-up. Workout sessions included 30 minutes of fitness and respiratory exercises, 30-minute specific respiratory exercises, 20-30 minute on cycle ergometer or treadmill at 30-80% of individual peak work rate, resistance exercise at 40-70% intensity of 1RM, 45 minutes of Nordic walking, and relaxation training.<br><br>CG (N=10): No exercise intervention. 6 weeks between initial and final assessment. Usual care with scheduled chemotherapy during Week 1 and 4.
Rutkowska et al., 2021[22]	IG (N=18): Personalized exercise regimens including warm-up. Workout sessions included 30 minutes of fitness and respiratory exercises, 30-minute specific respiratory exercises, 20-30 minute on cycle ergometer or treadmill at 30-80% of individual peak work rate, weighted exercise at 40-70% intensity of 1RM, 45 minutes of Nordic walking, and 20 minute Schultz autogenic training.<br><br>CG (N=8): No exercise intervention. 6 weeks between initial and final assessment. Usual care with scheduled chemotherapy during Week 1 and 4.
Cheung et al., 2021[23]	Aerobic intervention (N=10): aerobic exercise (30 mins of walking on treadmill, cycling on stationary bike at set pace tailored for moderate exercise) + strengthening exercise (30 mins - 4 strengthening exercises targeting arm, leg, abdomen with 10 reps of each exercise each time); encouraged to do to 90 mins/week self-practice (practice moderate-intensity aerobic exercise and to practice 2 sets of strengthening exercises with 10 repetitions each on alternate days) during intervention period<br><br>Tai Chi Intervention (N=9): 24-form Yang style of tai chi exercise set; session included warm up, relaxation in tai chi, cool down; encouraged to

	do to 90 mins/week self-practice (practice tai chi for 30 mins at least 3x per week) during intervention period<br><br>Self-Management Control Group (N=11):<br>Received written WHO exercise guidelines (stated to do at least 150 min of moderate intensity or 75 mins of vigorous aerobic exercise per week)<br><br>Standardized exercise log given to all participants to record during intervention.
Dhillon et al., 2017[24]	IG (N=56): 45 minutes of recreational aerobic physical activity + 15 minutes of behavior support sessions. Exercise was personalized to each participant's baseline fitness and interests.<br><br>CG (N=55): usual care, received general educational materials
Egegaard et al., 2019[25]	IG (N=8): 20 minutes of moderate to high intensity aerobic interval training on ergometer cycle (5-minute warm-up adjusted to 50-60% of individual peak power, three 5 minute exercise phases with 30 second break intervals); 1st and 3rd exercise phase at 80-90% individual peak power, 2nd phase at 80% individual peak power. Intensities increased over the 7-week course.<br><br>CG (N=7): No exercise training; work an activity tracker every date and rate their daily degree of side effects on record.
Hwang et al., 2012[26]	IG (N=13): 10-minute warm up followed by walking on treadmill or cycling on ergometer with 2-5 minute intervals alternating with high intensity (80% patients VO2peak or RPE (15-17)), and moderate intensity (60% VO2peak or RPE (11-13)). 5-minute cool down. Exercise regimen adjusted every 1-2 weeks.<br><br>CG (N=11): usual care, general patient education, social phone calls every 2-3vweeks with no supervised exercise intervention.
Kirca et al., 2021[27]	IG (N=42): Received all the same treatment as CG with the addition of relaxation exercises using MP3 (MP3 recording divided exercise into 10 minutes of relaxation description, 30 minutes of relaxation instruction, 30 minutes of music). Exercises included breathing control, clenching hands, flexing arms, raising eyebrows, clenching eyes, and contracting calves.<br><br>CG (N=42): Verbal education, educational booklet, standard nursing intervention, pharmacologic symptom management, weekly counseling phone calls for symptom management

Quist et al., 2020[28]	IG (N=110; at 12 weeks N=66): 10-minute warm up exercise (stationary cycling adjusted to 60-80% of patient HRmax), strength training (3 sets of 5-8 repetitions with 70-90% 1RM of leg press, chest press, lateral pull down, and leg extension), 10-15 minute aerobic training (interval training on stationary bikes at 70-90% HRmax), 5-10 minute stretching, and 15-20 minute progressive relaxation training<br><br>CG (N=108; at 12 weeks N=67): Usual care with recommendation to stay active
Zhang et al., 2016[29]	IG (N=48; analyzed N=47): 5–10-minute warm up followed by Yang style Tai Chi practice.<br><br>CG (N=48; analyzed N=44): low impact exercise group focused on arm, neck, and leg circles followed by upper and lower body stretches
Molassiotis et al., 2015[30]	IG (N=24): IMT delivered via pressure threshold device. Participants breathed in sufficiently forcefully enough to open the valve and allow for inhalation. Two treatments were completed at hospital and the remainder were done at home.<br><br>CG (N=23): Usual care, completed outcome measurements, received monthly home visits for assessment.<br><br>Baseline, T1= 4 weeks, T2 = 8, 12
Bade et al., 2021[31]	IG (n=20): 15 minute in-person teaching session about physical activity benefits, received FitBit Flex 2 accelerometer, individualized walking goals based on avg daily step count during 1st week, received twice daily gain-framed text messages. Encouraged to increase to 400 steps/day after each week (maximum 10,000 step/day recommendation). Prior week's recommendation is maintained if the goal is not met.<br><br>CG (n=20): usual care
Henke et al., 2014[32]	IG (N=18): Endurance training (walking in hallway for 6 minutes and stair walking exercise for 2 minutes). Intensity set between 55-70% of training HR. Strength training (3 sets with 1-minute breaks of abs, bicep curls, triceps extension with medium resistance band). Training set at 50% of individual max capacity. Includes active breathing exercises and conventional physiotherapy.<br><br>CG (N=11): Received conventional physiotherapy.
